# Using Antenna Arrays with Only One Active Element for Beam Reconfiguration and Sensitive Study in Dielectric Media

**DOI:** 10.3390/s21186019

**Published:** 2021-09-08

**Authors:** Borja Bayón-Buján, Aarón Ángel Salas-Sánchez, Juan Antonio Rodríguez-González, María Elena López-Martín, Francisco José Ares-Pena

**Affiliations:** 1 Department of Applied Physics, Faculty of Physics, University of Santiago de Compostela, E15782 Santiago de Compostela, Spain; borja.bayon@rai.usc.es (B.B.-B.); aaronangel.salas@usc.es (A.Á.S.-S.); ja.rodriguez@usc.es (J.A.R.-G.); 2 Department of Morphological Sciences, Faculty of Medicine, University of Santiago de Compostela, E15782 Santiago de Compostela, Spain; melena.lopez.martin@usc.es

**Keywords:** antenna arrays, mutual coupling, dielectric constant, beam reconfiguration

## Abstract

Antenna array pattern reconfiguration is usually achieved by changing the relative amplitudes and/or phases of the excitation distribution present in the array, at the cost of complex feeding networks. In this work, the mechanical displacement of a parasitic array perpendicular to another array with a single driven element is proposed. Additionally, the antenna is optimized addressing the variation of its response led by changes of the environmental dielectric constant of a surrounding gaseous medium. In such a way, a novel multipurpose antenna of utmost simplicity is obtained. From the computation of the self and mutual impedances, a control of the antenna radiation pattern by means of the induced currents in the parasitic elements is modelled. To illustrate the procedure, the technique will be applied to the variation of the side lobe level of a pencil beam and to obtain a flat-topped broadside beam from the same pencil beam, something with high interest for satellite applications. The proposed methodology represents an advance on the development of multipurpose antennas which resounds in simplicity not only in the reconfiguration of antenna beams, but in applications for the detection of particulate matter and/or measurements of the atmospheric dielectric constant.

## 1. Introduction

Reconfigurable antennas are radiating systems capable of modifying their properties dynamically, within a controlled strategy, and allowing reversibility. The reconfigurability of the antenna array, based on the modification of the geometry or behavior of its elements, maintains the efficiency of the antenna against changes on its environment and/or mission objectives. Moreover, reconfigurable antennas usually result in lower cost, weight, volume and complexity of maintenance and repair due to being able to offer the same functionalities as multiple conventional antennas [1].

However, the modification of the electrical characteristics of the antenna elements results in feeding networks of high complexity [2,3,4,5,6], where adding further controllable elements only worsens the situation. For this reason, the use of arrays of parasitic elements is very common: the currents of these elements are induced by near-field effects without the need of a feeding network of their own, allowing beam pattern reconfigurability with a notably much lower complexity [7,8]. Antennas making use of parasitic elements, commonly based around linear dipole arrays, range from the well-known Yagi-Uda antenna arrays [9,10] to more recent designs. In [8] the geometry of a parasitic linear dipole array in front of a fully driven one is changed to achieve pattern reconfiguration. In [7] linear and planar arrays are used toward the same goal by switching on and off some of the elements. In [11] printed dipole antennas with a single parasitic element are made capable of wide scanning angle with small deviation in antenna gain.

Asides from the interest in antenna beam pattern reconfiguration, differences in terms of response versus changes in the dielectric constant of the surroundings in which the antenna is immersed result in numerous applications. The relative dielectric constant of a gaseous medium mainly based on air is near unity, but, as is well-known, it varies with parameters as temperature, pressure and humidity [12,13], a fact that has already been found to be of use for hygrometry measurements using Yagi-Uda antennas [14]. By relating the changes in the environmental dielectric constant to changes in the variable of interest the latter can be measured. More critically, the constant can also vary in the presence of airborne particles [15]. From pollutants caused by urban and industrial contamination to water droplets in clouds, the detection and classification of such particles is more relevant than ever nowadays. For instance, the measurement of Particulate Matter (PM) has lately been receiving a lot of attention. Generally, it is measured using ground-based techniques [16], but satellite-based optical methods are used when non-local coverage is needed [17,18,19]. However, these methods make use of typically complex and expensive apparatus on top of not guaranteeing enough real-time resolution to follow on variable pollution scenarios. An alternative, the use of antennas measuring composition through its effects on the dielectric constant of the medium, was successfully studied in [20] using a linear array of shunt slots in the broad wall of an air-filled rectangular waveguide. A more recent study further developed the method using Yagi-Uda-like antennas [21], of low-cost construction and operation requirements.

Beyond PM detection, the ability to measure the dielectric constant of different types of media through light and mobile platforms is of interest for applications both on Earth and in planetary exploration. Changes in the dielectric constant of the atmosphere at different heights could lead to reflection and fading phenomena. Consequently, real-time measurement of these changes would allow for more efficient propagation of electromagnetic signals. In the case of the exploration of Titan by the Cassini spacecraft, for instance, ignoring the significant attenuation in radar data due to the presence of clouds could have led to misinterpretation of the abovementioned data [22]. Thus, the lack of certain knowledge of the abundance and composition of the materials in their respective surfaces and atmospheres serves as an impediment for the planning of future missions [23,24]. Furthermore, study of the dielectric constant gradient could also be of use to detect and study temperature inversion scenarios, due to their tendency to trap air masses with a noticeably different constant where the inversion occurs.

Therefore, in this work, a new antenna design based on linear dipole arrays with a single driven element is synthesized with the aim of obtaining an acceptable level of beam reconfigurability. Moreover, the antenna is further modified to maximize the variation of its response with a varying environmental dielectric constant to allow measurements or the detection of pollutants in the atmosphere. A novel multipurpose and efficient antenna of utmost simplicity is obtained.

## 2. Materials and Methods

### 2.1. The Antenna

**Figure 1:**
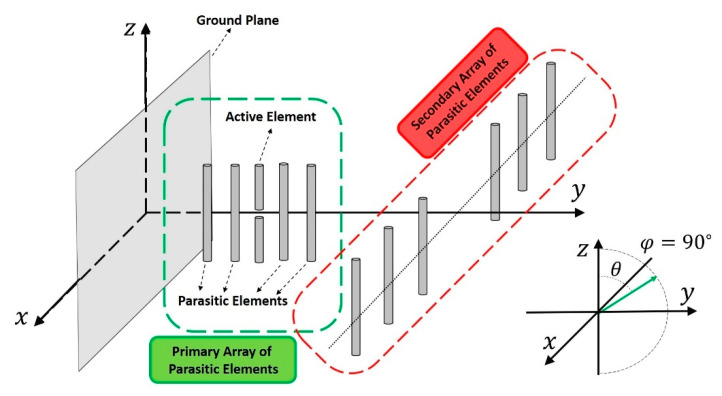
Sketch of the antenna proposed for exploiting the reconfiguration and the sensitivity to the dielectric constant.

**Figure 2:**
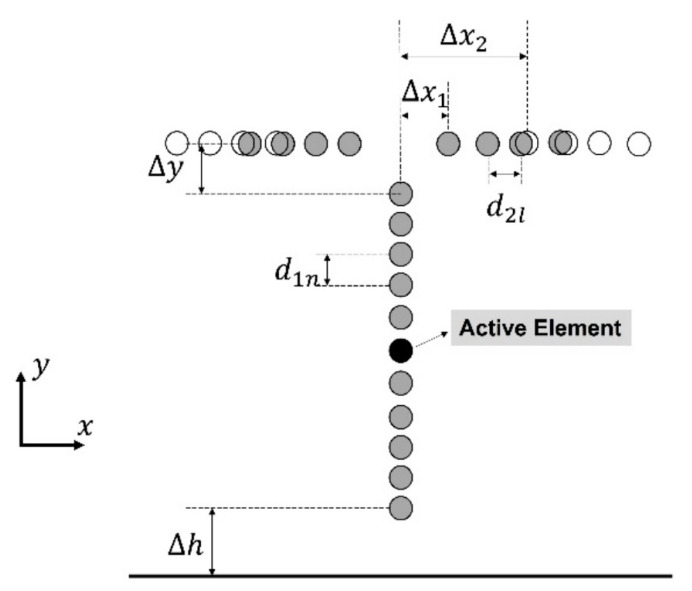
Geometrical description of the antenna array for performing the reconfiguration. The auxiliary array is represented in grey in a first configuration with central aperture $\Delta x_{1}$, and in white in a second configuration with central aperture $\Delta x_{2}.$ Generally $\Delta x_{2}\gg \Delta x_{1}$.

The antenna synthetized in this paper is made up of two linear dipole arrays, whose axis are perpendicular to each other (see Figure 1). The primary array, disposed by the y-axis, will be composed of a single driven dipole in its middle and an even number of parasitic dipoles surrounding it. The secondary or auxiliary array will be divided in two symmetric sections of parasitic dipoles and it will be displaced along the x-axis. As it is illustrated in Figure 2, antenna pattern reconfiguration is achieved by mechanically moving these already mentioned sections of the secondary array by using the same philosophy highlighted in [8]. To this aim, a simple mechanism as a rail and a step motor could be proposed. This procedure is provoked generally between a configuration close to the primary array and another one considerably further apart, where the primary array is practically unaffected by the auxiliary one. The antenna is assumed to be suspended in the air for modelling purposes.

Each dipole was modelled as a pair of hollow cylindrical conductors of very thin walls, length l and radius a, aligned in tandem with an infinitesimal gap between them. In the following it was assumed that 2l>>a, ka<<1, where k is the wavenumber, k=2π/λ. When a voltage is applied through the gap, a current distribution appears in the dipole, inducing a radiation.

The computation of the radiation far-field pattern and other parameters of interest requires the evaluation of the current distribution. In such a way, the relative currents were determined by including the mutual impedance data of the elements in the framework of a circuit-like equation. The magnitude and the phase of the currents in the parasitic elements depend on their sizes and spacings with respect to the driven element [25]. This can be accomplished by solving the system of equations resulting from applying transmission line theory to the antenna. Equivalently, the current amplitudes can be obtained via the matrix equation [V]=[Z][I]:

(1) $$\left(\begin{array}{c} V_{1}\\ \begin{array}{c} V_{2}\\ \ldots \\ V_{N} \end{array} \end{array}\right)=\left(\begin{array}{cccc} Z_{11} & Z_{12} & \ldots & Z_{1N}\\ Z_{21} & Z_{22} & \ldots & Z_{2N}\\ \ldots & \ldots & \ldots & \ldots \\ Z_{N1} & Z_{N2} & \ldots & Z_{NN} \end{array}\right)\cdot \left(\begin{array}{c} I_{1}\\ \begin{array}{c} I_{2}\\ \ldots \\ I_{N} \end{array} \end{array}\right)$$

where [V] is the known vector of voltages applied to the antenna elements (so $V_{i}=0$ for all elements but the driven one), [I] is the unknown vector of complex excitations and [Z] is the impedance matrix. The latter can be computed from the antenna characteristics (lengths, radii and relative separations of the elements) by means of the expressions given in [26] for the induced EMF method self-impedance and mutual impedances. Precisely, the appearance of induced currents in the parasitic elements is due to the presence of the off-diagonal terms, the mutual impedances.

The input impedance of the antenna can be obtained from the aforementioned matrix equation as well, being equal to:

(2) $$Z_{in}=Z_{kk}+\overset{N}{\underset{i\neq k}{\sum }}\frac{I_{i}}{I_{k}}\;Z_{ki}$$

The level of impedance matching between the antenna and the transmission line used to feed it can be formulated from the normalized difference between the input impedance ($Z_{in}$) and the characteristic impedance of the line ($Z_{0}$):

(3) $$\rho =\left|\frac{Z_{in}-Z_{0}}{Z_{in}+Z_{0}}\right|$$

which is the definition of the reflection coefficient of the antenna, that is, the ratio of the reflected wave to the incident wave. If the antenna is perfectly matched to a specific frequency $f_{0}$ and relative permittivity $\epsilon_{r}$, i.e., $Z_{in}=Z_{0},$ the reflection coefficient defined in (3) is equal to zero, which will be indispensable if an efficient antenna is to be designed.

As mentioned earlier, knowing the current distribution the radiation diagram F(θ,φ) can be computed as the sum of the radiating field contributions of each of the N antenna dipoles:

(4) $$F\left(\theta ,\varphi \right)=\overset{N}{\underset{i=1}{\sum }}I_{i}\;exp\left[jksin\theta \left(x_{i}cos\varphi +y_{i}sin\varphi \right)\right]f_{i}\left(\theta \right)$$

with the element factor $f_{i}\left(\theta \right)\;$given in [26], where N represents the summation of both the elements of the primary and auxiliary arrays, and $I_{i}$ is obtained by solving (1). The addition of a ground plane in the back of the antenna was modelled by means of the image principle, where this radiation system is equivalent to a pair of antenna arrays: the first real and the second one an array formed by virtual dipoles oppositely directed regarding to the first. The modified expressions of the impedances and element factor arising from the inclusion of the image dipoles are also given in [26]. In practice, the radiation pattern will be shown normalized to the main lobe maximum value, $F_{max},$ and expressed in dB, such that we will plot:

(5) $$F_{dB}\left(\theta ,\varphi \right)=20\;log_{10}\left(\left|\frac{F\left(\theta ,\varphi \right)}{F_{max}}\right|\right)$$

### 2.2. Measuring Polution: Effective Medium Model

**Figure 3:**
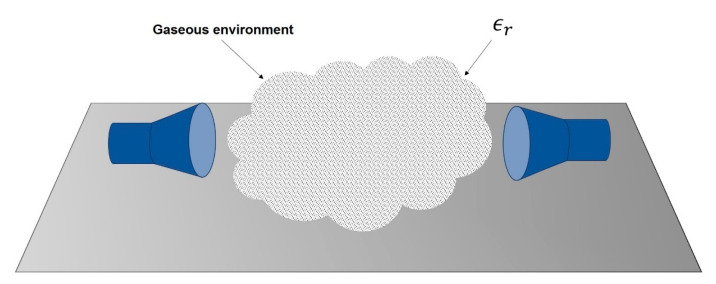
Working principle in presence of a gaseous medium.

The dielectric constant of a medium depends on the properties and abundance of its microscopic components. When electromagnetic radiation propagates in a medium with a dielectric constant $\epsilon_{r}$ (as sketched in Figure 3) its wavelength will be reduced as given by the following expression [21]:

(6) $$\lambda =\lambda_{0}/\sqrt{\epsilon_{r}}$$

where $\lambda_{0}\;$is the wavelength corresponding to $\epsilon_{r}=1.00$, and this will have an impact of the relative electric dimensions of the antenna. In such a way, the type and concentration of suspended matter in the air could be monitored; for instance, the modelling of particulate matter (PM) as spheres of dielectric constant $\epsilon_{i}$ with a volumetric concentration f was used in [15,21] to compute an effective dielectric constant of the medium.

**Figure 4:**
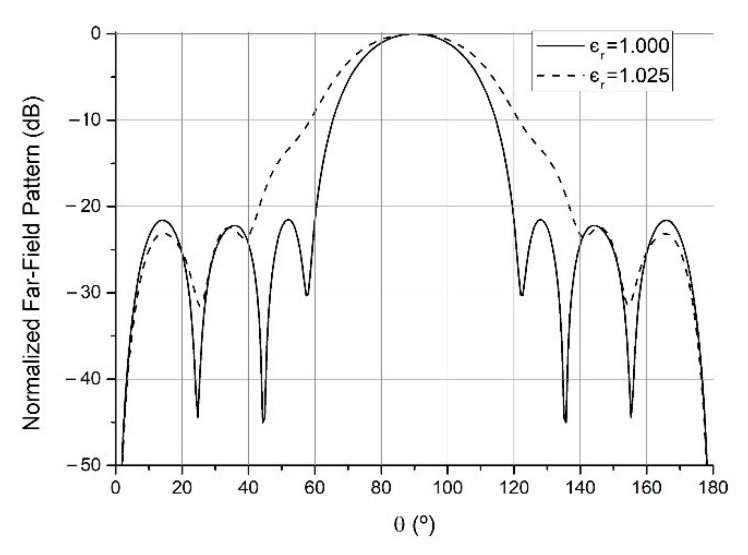
Effect of degradation versus $\epsilon_{r}\;$for far-field radiation patterns in the $\varphi =90^{{}^{\circ}}$ plane.

Assuming the antenna was matched in a vacuum, this growth necessarily leads to mismatch, so part of the received/transmitted energy is reflected, and the measured signal decreases, something which is measurable through the parameter ρ. On the other hand, the pattern itself will be degraded, generally in a way that causes the main lobe to widen and the minima to fill in (see Figure 4). This energy redistribution will lead to the variation of the absolute field magnitude F in every direction.

To sum up, as the basis of this effective medium model the addition of pollution to pure air is translated into changes in the dielectric constant of the medium, which leads to changes in antenna response. The characteristic curve describing these changes can be obtained from simulation or a process of calibration with particulate matter of known dielectric constant, so that it is possible to measure the environmental dielectric constant by keeping track of the variation of parameters like the reflection coefficient ρ or the field in a specific direction F(θ,φ). The changes in terms of dielectric constant will be referred to the real part of this parameter.

### 2.3. Numerical Simulation Algorithm

#### 2.3.1. Antenna Model and Optimization

A primary array of 11 elements (one driven, 10 parasitic) and auxiliary arrays of eight parasitic elements were used. The main reason behind these numbers was to achieve a comfortable reconfiguration capability while keeping a moderate boom length. Solutions with less elements were also tried, but without yielding promising results. A common radius of a=0.005λ was chosen, while the dipole lengths and relative separations were constrained in the optimization process to maintain the relevant approximations as is discussed in [26].

The primary array was first optimized to achieve a sum pattern with the lowest side lobe level (SLL) achievable. Afterwards, independent auxiliary arrays were added and optimized to achieve new radiation patterns or maximize the antenna response to changes in the dielectric constant of the medium. For the latter, two characteristic ranges of variation were chosen based on experimental effective dielectric constant measurements [21], from $\epsilon_{r}=1.000$ to $\epsilon_{r}=1.010$ and from $\epsilon_{r}=1.000$ to $\epsilon_{r}=1.025$, optimizing one independent auxiliary array for each range. Furthermore, one further auxiliary array was optimized for an antenna working on both ranges effectively, switching from one to the other by displacing the auxiliary array (varying its aperture).

#### 2.3.2. Optimization Strategy

Every result was synthetized using a common strategy written in the Python programming language, an open-source implementation of the widely used algorithm [27]. The strategy was based around the differential evolution (DE) [28] global optimization algorithm of the Scipy.optimize library. From an initial batch of candidate solutions, the algorithm creates new solutions based on the processes of mutation and recombination, choosing at the end the fittest solutions among the first and the new generations based in a given cost function. Iterating this process and assuming a proper choice of optimization parameters, the set of candidates will progress towards the global minimum of the cost function, and the optimization will end when a given tolerance or a maximum number of iterations is reached. The algorithm avoids getting stuck in local minima thanks to the use of multiple candidates in each iteration and the use of an stochastic distribution when defining parameters of the next generation.

The initial batch was randomly generated. The *best1bin* strategy was chosen: for each initial candidate a new one was created starting from the best candidate of the generation, $b_{0}$, and adding the difference between the vectors of two random candidates multiplied by a mutation factor:

(7) $$b^{\prime }=b_{0}+mutation\cdot \left(b\left[rand_{0}\right]-b\left[rand_{1}\right]\right)$$

where $rand_{0}$ and $rand_{1}\;$are two random indexes among those of the population. Starting from this trial vector and the initial candidate vector the next candidate is constructed. Each vector component is chosen based on a random number between 0 and 1 generated following a binomial distribution: if the number is below the recombination constant, the component is chosen from $b^{\prime }$; otherwise it is chosen from the initial candidate. Once the final candidate is finished its cost function is evaluated: if the cost is below that of the initial candidate, it will replace it in the next generation, otherwise the initial candidate remains. At the end of each iteration, when the next generation is fully obtained, the best candidate $b_{0}$ is updated, and the process is repeated.

The generic cost function used in every optimization, based on that of [7], was:

(8) $$cost\left(b\right)=a_{1}{\left(\Delta SLL\right)}^{2}+a_{2}{\left(\Delta ripple\right)}^{2}+a_{3}{\left(\Delta Z_{in}\right)}^{2}+a_{4}\frac{1}{{\left(\Delta \rho \right)}^{2}}+a_{5}\frac{1}{{\left(\Delta F\right)}^{2}}$$

where $a_{i}$ are coefficients chosen according to each goal of the optimization and the remaining parameters were defined as:

(9) $$\Delta SLL=SLL\left(b\right)-SLL_{desired}\;\Delta ripple=ripple\left(b\right)-ripple_{desired}\;\Delta Z_{in}=Z_{in}\left(b\right)-Z_{0}\;\Delta \rho =\rho \left(b,\epsilon^{\prime }\right)-\rho \left(b,\epsilon_{0}\right)\;\Delta F=F_{dB}\left(b,\epsilon^{\prime }\right)-F_{dB}\left(b,\epsilon_{0}\right)$$

$F_{dB}$ is the radiation field (4) in a specific direction, expressed in dB, and normalized to the value of the abovementioned field corresponding to $\epsilon_{0}:$ $F_{max}\left(\epsilon_{0}\right),$ such that and $\epsilon^{\prime }$ is the maximum value of ϵ in the working range. The *desired* parameters were to be defined at the start of each optimization. The b vector was constructed from the main antenna parameters: the length of the dipoles, the separation between them and their radius. After generating an initial candidate population, their corresponding impedance matrices were computed, from there the current excitation in each dipole, and, consequently, the antenna pattern was generated. Finally, all cost function parameters were obtained by computing the input impedance and measuring SLL and ripple from the antenna pattern. The algorithm was initiated several times for each set of objectives to choose the best result among those obtained, decreasing the chances of missing better solutions.

## 3. Results

### 3.1. Reconfiguration of the Antenna Beam

**Figure 5:**
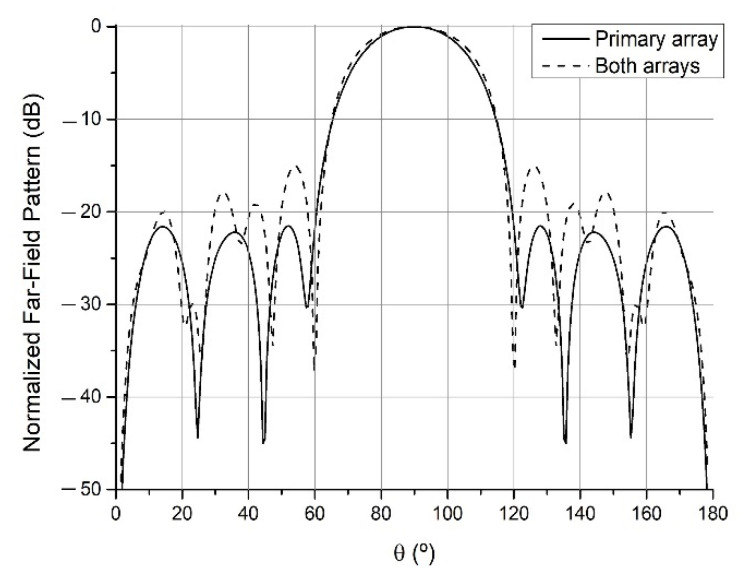
Far-field radiation patterns in $\varphi =90^{{}^{\circ}}$ plane generated by the primary array of Table 1 and the (a) array of Table 2, when the auxiliary array is sufficiently apart (“primary array” situation) and when it is close to the primary array (“both arrays” situation).

**Figure 6:**
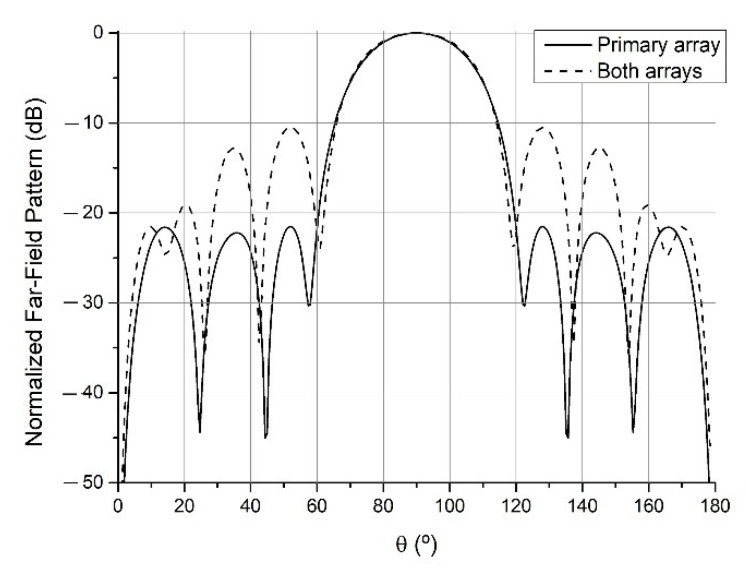
Far-field radiation patterns in $\varphi =90^{{}^{\circ}}$ plane generated by the primary array of Table 1 and the (b) array of Table 2, when the auxiliary array is sufficiently apart (“primary array” situation) and when it is close to the primary array (“both arrays” situation).

**Figure 7:**
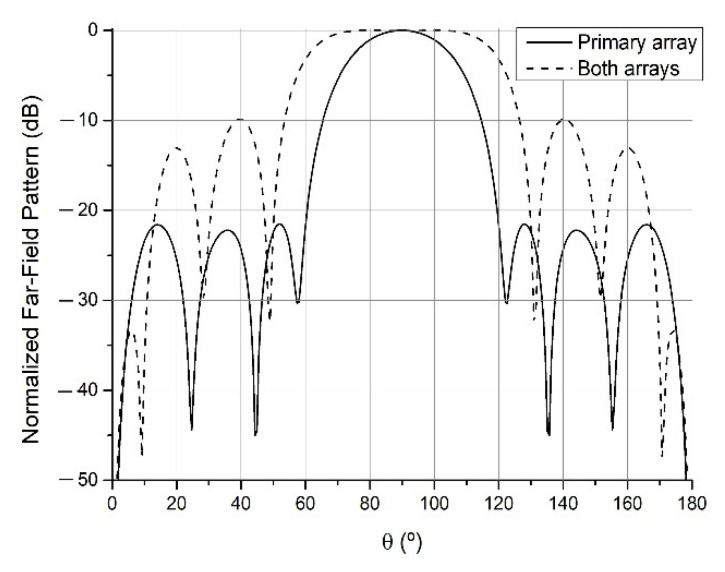
Far-field radiation patterns in $\varphi =90^{{}^{\circ}}$ plane generated by the primary array of Table 1 and the (c) array of Table 2, when the auxiliary array is sufficiently apart (“primary array” situation) and when it is close to the primary array (“both arrays” situation).

By this method, the primary array which geometry is depicted in Table 1 has been obtained. With the parameters set to $a_{1}=1,\;a_{2}=0,\;a_{3}=1,\;a_{4}=0,\;a_{5}=0,\;SLL_{desired}=-25\;\mathrm{dB}$ and $Z_{0}=\left(50+j0\right)\;\Omega$ the array radiated the pattern of the continuous lines of Figs. 5 to 7, a pencil beam with −21.5 dB of SLL. This will be the pattern recovered after the addition of different auxiliary arrays when they are displaced sufficiently apart, which will be denoted in the figures as the “primary array” situation.

Starting from this primary array, an auxiliary array was added and optimized keeping the previous selection of the parameters, except for $SLL_{desired}=-15\;\mathrm{dB}.$ This first added array can be found in the (a) column of Table 2, while the resulting antenna produced a pencil beam with −15.0 dB is shown in Figure 5. The same was done for $SLL_{desired}=-10\;\mathrm{dB},$ obtaining the auxiliary array corresponding to a pencil beam with −10.4 dB (see Figure 6). Finally, $SLL_{desired}=-10\;\mathrm{dB}$ was kept and $a_{2}=10,$ $ripple_{desired}=\pm 0.01\;\mathrm{dB}$ were set to obtain the auxiliary array resulting in the flat-topped beam pattern with a half-power beamwidth (width at −3 dB) of $57.3^{{}^{\circ}},$ ±0.02 dB of ripple and −9.0 dB of SLL of Figure 7. These last two arrays can also be found in Table 2. It is important to highlight that this reconfiguration has been performed in the cut corresponding to $\varphi =90^{{}^{\circ}}$ and $\theta \in \left[0,180^{{}^{\circ}}\right].$ Analysis regarding other different 2D cuts of the radiation pattern refers a degraded shape of the far-field.

### 3.2. Maximization of the Antenna Response to Changes in $\epsilon_{r}$

#### 3.2.1. Field in the Main Lobe Maximum

**Figure 8:**
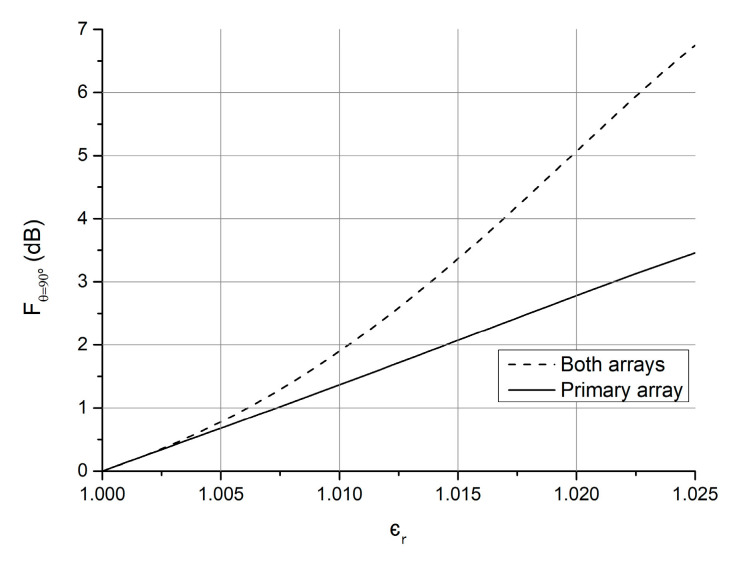
Field magnitude variation in the $\varphi =90^{{}^{\circ}}$, $\theta =90^{{}^{\circ}}$ direction of the primary array of Table 1 alone (“Primary array” situation) and with the addition of the auxiliary array of Table 3 with aperture $\Delta x_{1}$ (“Both arrays”).

**Figure 9:**
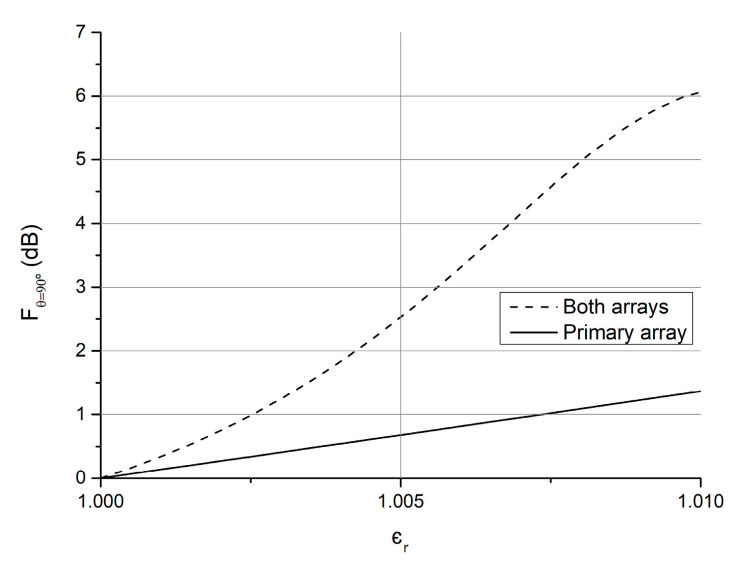
Field magnitude variation in the $\varphi =90^{{}^{\circ}}$, $\theta =90^{{}^{\circ}}$ direction of the primary array of Table 1 alone (“Primary array” situation) and with the addition of the auxiliary array of Table 3 with aperture $\Delta x_{2}$ (“Both arrays”).

The parameters were set to $a_{1}=0,\;a_{2}=0,\;a_{3}=1,\;a_{4}=0,\;a_{5}=1,$ focused in maximizing the variation of the field in the principal antenna direction, the main lobe maximum direction, $F_{dB}\left(\theta =90^{{}^{\circ}},\varphi =90^{{}^{\circ}}\right).$ Starting from the primary array synthetized in the previous section, the auxiliary array of Table 3 was added to achieve the characteristic curve of Figure 8 when the array is kept displaced from the primary one by $\Delta x_{1}=1.1721$, and that of Figure 9 when it is displaced by $\Delta x_{2}=6.0031.$ Comparing the response obtained with the whole antenna to that of the primary array alone, an increase of 3.2 dB in $\epsilon_{r}=1.025$ and of 4.7 dB in $\epsilon_{r}=1.010$ were obtained with the respective displacements.

Further auxiliary arrays were optimized to work efficiently in each range, and the results were similar to the multirange array. An increase of 2.5 dB in $\epsilon_{r}=1.025$ and of 4.3 dB in $\epsilon_{r}=1.010$ were obtained with the independent auxiliary arrays.

#### 3.2.2. Reflection Coefficient

The optimization parameters were set to $a_{1}=0,\;a_{2}=0,\;a_{3}=1000,\;a_{4}=1,\;a_{5}=0,$ so the focus was shifted to a maximization of the variation of the reflection coefficient. Starting once more with the same primary array, a first attempt with the multirange approach allowed an increase of 0.004 in $\epsilon_{r}=1.025$ and of 0.084 in $\epsilon_{r}=1.010$. An attempt with independent auxiliary arrays for each range yielded better results: an increase of 0.037 dB in $\epsilon_{r}=1.025$ and of 0.157 in $\epsilon_{r}=1.010$. These results are similar to the obtained for antenna arrays with similar dipole length characteristics referred in [21].

## 4. Discussion

In this work, an antenna design capable not only of achieving reconfiguration of the antenna beam with the linear displacement of some of its elements, but also of presenting an improved sensitivity to changes in the dielectric constant of its surroundings was presented. By proper choice of an appropriate auxiliary array the antenna can swiftly change from one function to the next. This reconfigurability and multifunctionality was all achieved with a single active element, simplifying the feeding network of the antenna to its most basic form on top of keeping a low power consumption, especially when compared to other types of reconfigurable antennas. The results related to the sensitivity of the antenna to changes in dielectric constant as seen in the variation of the absolute field value in a certain direction were indeed promising, as was the design for an antenna able to operate in more than one range of variation efficiently.

In practice, the auxiliary array displacement could be implemented by means of a simple mechanical system consisting in a rail and a micro-step motor. For the measuring of changes in the absolute field value emitted or received in the main lobe another antenna would be needed in the far-field. If this study were to be undertaken in the near field the calculations shown here would have to be modified to account for this and the effect of the second measuring antenna on the field of the first.

Regarding future developments, it would be interesting to extend the study to other shapes of the antenna beam. Furthermore, it is worth highlighting that, while in the present work it was chosen to keep the same primary array as the basis for all the antennas synthesized, the array in question was optimized to yield a low SLL, and as such its response to changes in the dielectric constant of the medium was not optimal. Follow-up studies could start from a primary array optimized to maximize the change in antenna response or optimized using the whole cost function to find a middle point between the two uses of interest. Again, the addition of auxiliary arrays would guarantee the ability to further tailor the antenna to the application in mind. Finally, it could also be interesting to add more elements to each array for applications were a few more dBs of SLL would be worth the increase in boom length.

## Figures and Tables

**Table 1:** Geometrical description of the primary array antenna with one active element. The input impedance of the array is also shown.

Element No.	$l_{n}\left(\lambda \right)$	$d_{n}\left(\lambda \right)$
1	0.2399	−
2	0.2164	0.3595
3	0.2091	0.2337
4	0.2388	0.4339
5	0.2156	0.1724
6 ^a^	0.2401	0.2441
7	0.2272	0.3615
8	0.2392	0.2558
9	0.2297	0.2066
10	0.2460	0.3982
11	0.2159	0.2279
Δh(λ)	0.1118	
$Z_{in}\left(\Omega \right)$	49.82+j0.13	

^a^ Active element.

**Table 2:** Geometrical description of one of the symmetrical halves of each auxiliary array added to reconfigure the original pencil beam to (a) a pencil beam with −15 dB of SLL (b) a pencil beam with −10 dB of SLL (c) a flat-topped beam. The input impedances of the corresponding antennas (with both the primary and corresponding auxiliary arrays) are also shown.

	(a)		(b)		(c)	
**Element No.**	$l_{n}\left(\lambda \right)$	$d_{n}\left(\lambda \right)$	$l_{n}\left(\lambda \right)$	$d_{n}\left(\lambda \right)$	$l_{n}\left(\lambda \right)$	$d_{n}\left(\lambda \right)$
1	0.2537	0.2769	0.2651	0.4936	0.2197	0.3273
2	0.2464	0.3990	0.2366	0.1606	0.2561	0.5000
3	0.2678	0.1340	0.2163	0.4924	0.2414	0.1000
4	0.2640	−	0.2609	−	0.2280	−
Δx(λ)	1.3443		1.1271		0.2082	
Δy(λ)	0.3281		−0.8085		−0.8467	
$Z_{in}\left(\Omega \right)$	49.98+j0.08		50.20+j0.25		50.3+j0.04	

**Table 3:** Geometrical description of the auxiliary array added to maximize the change of the field magnitude in the main lobe direction in two ranges of variation of the dielectric constant of the surroundings: $\Delta x_{1}\;$is the displacement for $\epsilon_{r}\in \left[1.000,\;1.010\right],$ while $\Delta x_{2}$ is for $\epsilon_{r}\in \left[1.000,\;1.025\right].$

Element No.	$l_{n}\left(\lambda \right)$	$d_{n}\left(\lambda \right)$
1	0.2669	0.1925
2	0.2216	0.2277
3	0.2282	0.2412
4	0.2430	-
Δy(λ)	−0.9992	
$\Delta x_{1}\left(\lambda \right)$	1.1721	
$\Delta x_{2}\left(\lambda \right)$	6.0031	

## Data Availability

Data is contained within the article.
